# Human embryos in a dish – modeling early embryonic development with pluripotent stem cells

**DOI:** 10.1186/s13619-022-00107-w

**Published:** 2022-01-14

**Authors:** Xiukun Wang, Guang Hu

**Affiliations:** Epigenetics and Stem Cell Biology Laboratory, National Institute of Environmental Health Sciences Research Triangle Park, Durham, NC 27709 USA

**Keywords:** Pluripotent stem cells, Stem cell-based embryo models, Blastoid

## Abstract

Stem cell-based embryo models present new opportunities to study early embryonic development. In a recent study, Kagawa et al. identified an approach to create human pluripotent stem cell-based blastoids that resemble the human blastocysts. These blastoids efficiently generated analogs of the EPI, TE, PrE lineages with transcriptomes highly similar to those found in vivo. Furthermore, the formation of these lineages followed the same sequence and pace of blastocyst development, and was also dependent on the same pathways required for lineage specification. Finally, the blastoids were capable of attaching to stimulated endometrial cells to mimic the process of implantation. While more comprehensive analysis is needed to confirm its validity and usefulness, this new blastoid system presents the latest development in the attempt to model early human embryogenesis in vitro.

## Main Text

Understanding human embryonic development can provide important insights to both basic research and clinical applications. However, it is difficult to study the natural development of human embryos for ethical and technical reasons, and it is also challenging to carry out experiments in vitro due to the lack of embryonic materials. As an alternative, stem cell-based embryo models have opened an unprecedented avenue for modeling embryogenesis. For example, mouse blastocyst-like structures have been generated using various pluripotent stem cells (PSCs) or a mixture of pluripotent and extraembryonic stem cells. These structures form by self-organization under the appropriate culture conditions and recapitulate many of the early developmental events (reviewed in Posfai et al. 2021; Rossant and Tam 2021)). More recently, human stem cell-based embryo models have also been created (Fan et al. 2021; Liu et al. 2021; Sozen et al. 2021; Yanagida et al. 2021; Yu et al. 2021), but the fidelity to which the human models mimic blastocyst development has been challenged (Zhao et al. 2021).

In a recent issue of Nature, Kagawa et al. reported a new approach to generate human blastocyst-like structures in vitro using naïve human PSCs (Kagawa et al. 2021). These naïve human PSCs are derived from the inner cell mass cells in the blastocysts or the primed PSCs using culture conditions that block various differentiation-inducing pathways (reviewed in (Pera and Rossant 2021)). Compared to conventional human PSCs that are maintained in the primed state, they display a broader developmental potential and can readily differentiate into the trophectoderm lineage upon induction (Guo et al. 2021; Io et al., 2021). To initiate blastocyst-like structure formation, the authors aggregated the naïve human PSCs in non-adherent hydrogel microwells, and cultured them in the presence of a combination of inhibitors and cytokines of key pathways regulating the specification of epiblast (EPI) and trophectoderm (TE) lineages. Specifically, they used the Hippo and TGFβ pathway inhibitors to promote TE formation, the ERK inhibitor and LIF to support the EPI fate, and the ROCK inhibitor to presumably reduce cell death. Under this condition, the cells efficiently and consistently self-organized to form blastocyst-like structures or blastoids. The blastoids contain a TE-like outer shell with apical-basal polarity and tight junctions, which expresses TE markers. They also have inner cell mass-like cell clusters that can further develop into EPI-like cells and primitive endoderm (PrE)-like cells. Importantly, the authors showed that only the naïve but not primed human PSCs can form the blastoids, suggesting that the broad developmental potential of the naïve PSCs is critical for this process.

Next, the authors validated the identities of the cells in the blastoids by single-cell and bulk RNA sequencing. Based on gene expression profiles, the blastoid cells were found to mainly exist in three distinct states that are largely similar to the EPI, TE, and PrE lineages in normal blastocysts. Notably, unlike previous attempts, the blastoid cells can be clearly distinguished from cells isolated from the post-implantation stage embryos. Functionally, the blastoids can give rise to naïve human PSCs and TSCs, and the derived naïve PSCs can again generate 2^nd^ blastoids. In addition, the specification and morphogenesis of TE-like cells in the blastoids are dependent on atypical PKC and Hippo signaling, two pathways known to be critical in TE differentiation. Furthermore, the sequence of lineage marker expression and the appearance of the EPI, TE, PrE lineages follow the same order as observed in normal blastocyst development. Finally, the authors tested how the blastoids can be used to model implantation in vitro. They first developed a 2D open-faced endometrial layer (OFEL) system that responds to hormones in a similar fashion as the uterine endometrium at the time of implantation. The authors found that human naïve PSC-based blastoids only attached to the hormone-stimulated OFEL cells but not the un-stimulated ones, and the attachment occurs at the polar TE region of the blastoids and requires the presence of the EPI-like cells. After attachment to OFEL, the EPI-, TE-, PrE-like cells continued to expand upon prolonged culture. The TE-like cells formed trophoblasts expressing chorionic gonadotropin β, and further differentiated into syncytio- and extravillous trophoblasts. The EPI-like cells maintained pluripotency gene expression but up-regulated primed and epithelialization markers. Therefore, the blastoids and OFEL system may indeed be used to uncover new molecular details in peri- and post-implantation events.

Blastoids can help to uncover the developmental trajectories and milestones in early embryonic development, such as compartmentalization, lineage segregation, and implantation. Previous human PSC-based blastoid models were not able to recapitulate all the features of the developing blastocysts. Specifically, although blastocyst-like structures have been created from human expanded pluripotent stem cells, induced pluripotent stem cells, or naïve pluripotent stem cells cultured in a different condition, they showed significant divergence from natural blastocysts in marker expression, signaling pathways, cell composition, and cell identity (Sozen et al. 2021), especially in the TE lineage (Zhao et al. 2021). These limitations put significant constraints on their utility for modeling embryogenesis. The current study made great strides by using a naïve human PSC model with demonstrated developmental potentials toward TE, and by optimizing culture conditions to support the expansion of both the EPI and TE lineages (for comparison across the methods, please see Table 1). As a result, the blastoids generated in this study appeared to better approximate normal blastocysts in cell type constitution, and the EPI-, TE-, and PrE-like cells also seem to show better similarity to their in vivo counterparts. As TE is essential for implantation and epiblast patterning during gastrulation, this new blastoid model may thereby provide a better tool to study early human development. In addition, the successful formation of blastocyst-like structures using only human naïve PSCs supported the notion that the paradigm of early lineage segregation is different between human and mouse. Human pre-implantation epiblast cells and cultured PSCs were found to retain the ability to generate trophoblasts (Guo et al. 2021; Io et al. 2021). In contrast, mouse PSCs are restricted from differentiating into TE, and mouse PSC-based blastoids can only mimic germ layer specifications but not replicate the cell lineages in the blastocysts (Rossant and Tam 2021). Together, this study highlighted the importance in the choice of the starting cell type(s) and culture conditions in the stem-cell based human embryo model.

**Table 1:** Human stem cell-based embryo models

Reference	Starting cells	Culture media	Culture condition	Blastoids formation
Fan et al. (2021)	hEPS in LCDM + hEPS-derived TE-like cells	IVC1, IVC2	AggreWell	6 days
Liu et al. (2021)	hiPSC in t2iLGoY	iBlastoid	AggreWell	6 days
Yu et al. (2021)	Naïve hPSC in 5i/L/A or PXGL	HDM, TDM	AggreWell	9 days
Yanagida et al. (2021)	Naïve hPSC in PXGL	PD + A83 + Y, A83, N2B27	U-bottom 96-well	3 days
Sozen et al. (2021)	hEPS in hEP	IVF + hEP + hTS (5% O_2_)	AggreWell	6 days
Kagawa et al. (2021)	Naïve hPSC in PXGL	N2B27 + Y, PALLY, LY	Non-adherent hydrogel microwell	4 days

LCDM: N2B27 with 10 ng/ml human LIF, 3 μM CHIR99021, 2 μM (S)-( +)-Dimethindene maleate and 2 μM minocycline hydrochloride, 1 μM IWR endo-1, and 2 μM Y-27632

IVC1: Advanced DMEM/F12, 20% Heat-inactivated FBS, 2 mM L-glutaMAX, 0.5% penicillin/streptomycin, 1% ITS-X, 1% sodium pyruvate, 8 nM β-estradiol, 200 ng/mL progesterone and 25 μM N-acetyl-L-cysteine

IVC2: Advanced DMEM/F12, 30% Knockout serum, 2 mM L-glutaMAX, 0.5% penicillin/streptomycin, 1% ITS-X, 1% sodium pyruvate, 8 nM β-estradiol, 200 ng/mL progesterone, 2 μM Y27632 and 25 μM N-Acetyl-L-cysteine

t2iLGoY**:** 50:50 mixture of DMEM/F-12 (ThermoFisher) and neurobasal medium (ThermoFisher), supplemented with 2 mM L-glutamine (ThermoFisher), 0.1 mM 2-mercaptoethanol (ThermoFisher), 0.5% N2 supplement (ThermoFisher), 1% B27 supplement (ThermoFisher), 1% penicillin–streptomycin (ThermoFisher), 10 ng/ml human leukaemia inhibitory factor (LIF, made in house), 250 μM L-ascorbic acid (Sigma), 10 μg/ml recombinant human insulin (Sigma), 1 μM PD0325901 (Miltenyi Biotec), 1 μM CHIR99021 (Miltenyi Biotec), 2.5 μM Gö6983 (Tocris), and 10 μM Y-27632 (ROCK inhibitor; Selleckchem)

iBlastoid: 50:50 mixture of DMEM/F-12 (ThermoFisher) and neurobasal medium (ThermoFisher), supplemented with 2 mM l-glutamine (ThermoFisher), 0.1 mM 2-mercaptoethanol (ThermoFisher), 0.5% N2 supplement (ThermoFisher), 1% B27 supplement (ThermoFisher), and 1% penicillin–streptomycin (ThermoFisher))

5i/L/A: N2B27 basal medium with 1 × GlutaMAX (Gibco), 1 × nonessential amino acids (Gibco), 0.1 mM β-mercaptoethanol (Gibco), 0.5% penicillin–streptomycin (Gibco), 50 mg/ml bovine serum albumin (BSA, Sigma) and the following small molecules and cytokines: 1 μM PD0325901 (Stemgent), 0.5 or 1 μM IM-12 (Enzo), 0.5 μM SB590885 (R&D systems), 1 μM WH-4–023 (A Chemtek), 20 ng/ml recombinant human LIF (Peprotech) and 10 ng/ml activin A (Peprotech). PXGL medium: N2B27 basal medium with 1 × GlutaMAX, 1 × nonessential amino acids, 0.1 mM β-mercaptoethanol, 0.5% penicillin–streptomycin and the following small molecules and cytokines: 1 μM PD0325901, 2 μM XAV939 (Sigma), 2 μM Go6983 (Sigma) and 20 ng/ml recombinant human LIF

HDM: 1:1 (v/v) mixture of DMEM/F12 and neurobasal medium, 1 × N2 supplement, 1 × B27 supplement, 1 × GlutaMAX, 1 × nonessential amino acids, 0.1 mM β-mercaptoethanol, 0.5% penicillin–streptomycin, 20 ng/ml bFGF (Peprotech), 20 ng/ml activin A and 3 μM CHIR99021

TDM: 1:1 (v/v) mixture of DMEM/F12 and neurobasal medium, 0.5 × N2 supplement, 0.5 × B27 supplement, 0.5% ITS-X, 0.5 × GlutaMAX, 0.5 × nonessential amino acids, 0.1 mM β-mercaptoethanol, 0.5% knockout serum replacement (KSR, Gibco), 0.1% FBS, 50 mg ml − 1 BSA, 0.5% penicillin–streptomycin, 1 μM PD0325901, 0.5 μM A83-01, 0.25 μM SB590885, 0.5 μM WH-4–023, 0.25 μM IM-12, 1 μM CHIR99021, 0.5 μM SB431542, 10 ng ml − 1 recombinant human LIF, 25 ng ml − 1 EGF, 0.75 μg ml − 1 l-ascorbic acid and 0.4 mM VPA. For certain experiments the following chemicals were added to the TDM: 100 nM Go6976 (Selleckchem), 1 μM Go6983 (Selleckchem), 10 μM PKCα (C2-4) inhibitor peptide (Cayman), 10 μM PKCζ pseudosubstrate inhibitor (Cayman), 10 μM PKCη pseudosubstrate inhibitor (Sigma) or 10 μM PKCδ inhibitor KAI-9803 (delcasertib hydrochloride, MedChemExpress)

PD + A83 + Y: N2B27 supplemented with 1.5 mM PD0325901, 1 mM A83-01 and 10 mM Y-27632

A83: N2B27 supplemented with 0.5 mM A83-01

N2B27 + PALLY: N2B27 supplemented with PD0325901 (1 μM), A 83–01 (1 μM, MedChemExpress, HY-10432), 1-Oleoyl lysophosphatidic acid sodium salt (LPA)32 (500 nM, Tocris, 3854), hLIF (10 ng/ml), and Y-27632 (10 μM)

hEP: N2B27 supplemented with 10 ng/ml recombinant human LIF (L, 10 ng/ml; Peprotech, 300–05), CHIR99021 (C, 1 mM; Stem Cell Technologies), (S)-( +)-Dimethindenemaleate (D, 1 mM; Tocris, 1425) and Minocycline hydrochloride (M, 2 mM; Santa Cruz Biotechnology, sc-203339), Y-27632 (5 µM)

IVF + hEP + hTS: 50% IVF media (Continuous Single Culture-NX Complete (CSCM-NXC)) (90,168, FUJIFILM), 25% hEP media, and 25% hTS media, supplemented with CHIR99021 (2 μM), Y27632 (5 μM), BMP4 (20 ng/mL), FGF2 (40 ng/mL), and A83-01 (2 μM). After 48 h, FGF2 is lowered to 20 ng/mL and A83-01 is omitted

## Data Availability

Not applicable

## Abbreviations

PSC: Pluripotent stem cells
EPI: Epiblast
TE: Trophectoderm TSC Trophoblast stem cells
TSC: Trophoblast stem cells
PrE: Primitive endoderm
OFEL: Open-faced endometrial layer
